# Emotion in the Area of Entrepreneurship: An Analysis of Research Hotspots

**DOI:** 10.3389/fpsyg.2022.922148

**Published:** 2022-06-15

**Authors:** Xifeng Lu, Yiyu Xiong, Xingqun Lv, Biaoan Shan

**Affiliations:** ^1^College of Accounting, Jilin University of Finance and Economics, Changchun, China; ^2^School of Business and Management, Jilin University, Changchun, China; ^3^School of Entrepreneurship Education, Heilongjiang University, Harbin, China; ^4^Post-doctoral Research Station of Law, Heilongjiang University, Harbin, China

**Keywords:** emotion, entrepreneurship, entrepreneurial passion, research hotspots, bibliometric analysis

## Abstract

The application of emotion in economic management is gaining attention. As an important irrational factor, personal emotion often plays a significant role in business decision-making activities. In the field of entrepreneurship, emotion also plays a crucial role, and more and more scholars are focusing on this interdisciplinary issue. However, the current research on emotion in entrepreneurship is still fragmented, and there is an urgent need for a more scientific and systematic approach to comprehensively organize the literature in this field, so as to lay the foundation for researchers to further research on emotion in entrepreneurship. In this study, VOSviewer was used to analyze the existing literature, and the results showed that the current research on emotion in the field of entrepreneurship mainly focuses on five research themes, namely, emotion and college students' entrepreneurship, family emotion and entrepreneurship, the role of emotion in successful entrepreneurship, emotional problems under the influence of entrepreneurial failure, and entrepreneurial passion.

## Introduction

The application of emotion in economic management has attracted more and more attention. As an important irrational factor, personal emotion often plays a significant role in enterprise decision-making activities. Especially in the field of entrepreneurship, previous studies have found that individual emotional factors, including psychological variables such as passion and wellbeing, play a more and more important role in the entrepreneurial decision-making process (e.g., Cardon et al., 2009; Davis et al., 2017; Dijkhuizen et al., 2018). Some very successful entrepreneurs in the world, such as Musk, Jeff Bezos, Zuckerberg and so on, have founded extremely successful enterprises, and they often reflect rich personal emotions such as passion. More and more evidence shows that these diversified affective factors, along with the process of identification, evaluation and utilization of entrepreneurial opportunities, become the key antecedents affecting entrepreneurial success (e.g., Miller et al., 2012; Kollmann et al., 2017; Allen et al., 2021).

However, for a long time in the past, the role of emotional factors has been ignored by researchers in the field of entrepreneurship. The previous literature has found and paid attention to the significant impact of individual characteristics on entrepreneurial activities for a long time. For example, the researchers explored the influence of personality traits (such as big five traits), cognitive factors (such as illusion of control, experiential learning), social capital and human capital of entrepreneurs or senior managers on the formation of entrepreneurial intention, the identification and development of entrepreneurial opportunities, resource acquisition and integration (e.g., Ciavarella et al., 2004; Unger et al., 2011; Henley et al., 2017). A series of research results have been formed around individual characteristics. However, as the entrepreneurial process and mechanism are revealed step by step, the uncertainty of entrepreneurial activities and the role of individual irrational factors (emotion) are gradually excavated. Through empirical and experimental studies, some research literatures have found that individual emotion helps to deal with various uncertainties in the entrepreneurial process, and is an important determinant of entrepreneurial decision-making.

Therefore, in recent years, the application of emotion in the field of entrepreneurship has received great attention. Scholars classify emotion to reveal the role of entrepreneurial passion, positive or negative emotion, optimism and other emotional characteristics in the process of entrepreneurial opportunity development or resource development (e.g., Wang et al., 2016; Hubner et al., 2020; Haddoud et al., 2022). Psychological analysis methods, such as experimental research, quasi-experimental research and EEG analysis, have been applied to the study of emotional content in the field of entrepreneurship. These research results have greatly enriched the traditional theoretical system of entrepreneurship. At the same time, the relevant research findings also supplement that we only focused on the rational side of entrepreneurial activities in the past while ignoring the influence of irrational emotional factors. This provides theoretical support for researchers to fully understand the process and mechanism of entrepreneurial activities.

With the continuous development of emotional research, it is gratifying that more and more scholars begin to join in the study of this interdisciplinary problem. However, the current research literature is still relatively decentralized. Therefore, there is an urgent need for more scientific and systematic methods to comprehensively sort out the literature in this field, so as to lay a foundation for researchers to further explore emotional problems in the field of entrepreneurship. Based on this, this paper intends to use the bibliometric analysis method, with the help of VOSviewer software to sort out the emotional research literature in the field of entrepreneurship. The contribution of this research mainly includes two aspects. Firstly, we intuitively analyze the emotional research in the area of entrepreneurship by using scientific bibliometric analysis method. We use the VOSviewer software to build a research topic framework to help researchers understand this field more comprehensively. Secondly, we analyze the shortcomings of existing research and point out some feasible directions for future research.

Through literature analysis, this study attempts to answer the following key questions: (1) What are the characteristics of the existing emotional research in the field of entrepreneurship in terms of quantity, periodicals and country/region distribution? (2) What is the situation of co-creation by authors in this field? (3) What are the current research hotspots in this field? For this purpose, the structure of this paper is as follows. Firstly, the data sources, data processing and analysis tools are introduced; Secondly, the existing research is analyzed as a whole; Then the five research hotspots of emotion research in the field of entrepreneurship are summarized; Finally, the conclusion.

## Methodology

Using bibliometric method, we choose VOSviewer as our analysis software, so as to study emotion research in entrepreneurial field more scientifically. At the same time, we conducted a comprehensive analysis of the emotional research in entrepreneurship area, including the quantitative distribution, periodicals distribution, country/regional distribution, current research hotspots and future research directions. Bibliometric technology is based on bibliometric data. Compared with traditional literature analysis methods, it is more systematic and transparent (Wörfel, 2021), and can better help us understand the distribution, dynamic trend, key points and future research direction of knowledge network (Baier-Fuentes et al., 2019; Zaheer et al., 2019; Velt et al., 2020).

### Data Sources

The data used in this study come from the core collection of web of science (WoS), because the WoS database covers a wide range of outstanding journals in various academic fields, and we can find accurate author, journal, publication date, keyword and other information that can be used for analysis (Ellegaard and Wallin, 2015; Mongeon and Paul-Hus, 2016; Ye et al., 2020) in the database. In order to ensure the authoritative and scientific nature of the article, we limit the literature type to “article”. At the same time, as most of the emotional studies in the field of entrepreneurship are written in English, and in order to ensure the readability of the retrieved articles, we limit the language to English. In order to make our data contain all the literature related to the topic as much as possible, we finally determine that the search language is TS= (emotion^*^) AND TS= (entrepreneur^*^) AND DT= (Article) AND LA= (English). Through the search, we obtained 901 related articles on March 20th, 2022.

### Data Screening

In order to ensure that the final literature used for analysis is strongly related to our topic, we have read the titles, keywords, keywords plus and abstracts of 901 articles. For some still uncertain literature, we read the introduction and conclusions or the full text. After three rounds of screening, we excluded some articles, including:

(1) Repetitive articles and articles that are unable to obtain the original text.
(2) The content of the article is only related to entrepreneurship, not related to emotion, or very little mention of emotion.
(3) The content of article deals with the practice of emotion in management, but it does not involve entrepreneurship or new enterprises.
(4) The article belongs to the fields of medicine or physics, and has nothing to do with business economics.

Finally, we got 479 articles for bibliometric analysis.

### Analytical Tools

Lots of scolars use professional bibliometric tools to visually analyze the research hotspots, such as VOSviewer, CiteSpace, SciMAT and so on. In this study, VOSviewer software is used as the research hotspot analysis tool because it is the most frequently used software in bibliometric research in various fields (Pan et al., 2018). VOSviewer is a computer program (van Eck and Waltman, 2010) that can be used to create visual econometric maps of scientific literature. It can analyze all kinds of bibliometric network data, such as the citation relationship between publications or periodicals, the collaborative relationship among scholars and the co-occurrence relationship among various terms (van Eck and Waltman, 2017). Using software, we make a co-author analysis of the emotional research in the entrepreneurial field, and use the term co-occurrence function of VOSviewer to cluster the existing research in this field.

## Results

### Quantitative Distribution

**Figure 1:**
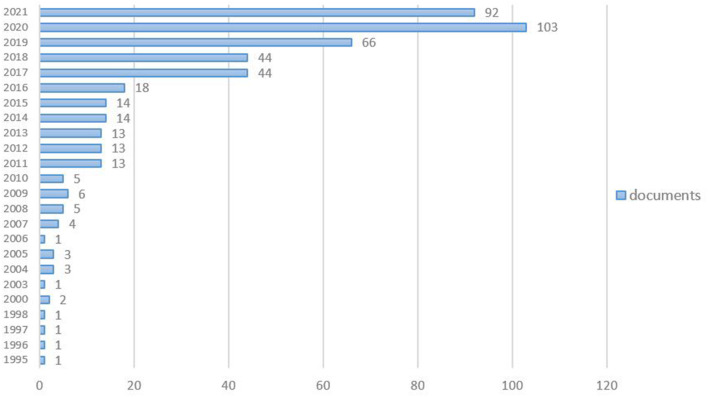
Distribution of research quantity.

We analyze the quantitative distribution of emotional research in the area of entrepreneurship (as shown in Figure 1) and find that the number of such studies as a whole is gradually increasing. In 1995, some scholars realized that the emotion of entrepreneurs had an impact on their entrepreneurial results, but until 2010, the level of academic attention in this field was not high. Between 2011 and 2016, the number of emotional research results in entrepreneurship increased steadily. The number of research in this field surged in 2017 and continued to grow in the following years, reaching a peak in 2020, with the number of studies exceeding 100. The existing emotional research in the field of entrepreneurship has been published since 2017, so this field is a new research field. With the increasing number of emotional research in the field of entrepreneurship, there is an urgent need to use scientific analysis software to systematically sort out and classify the existing research.

### Periodicals Distribution

As shown in Table 1, the top five publications published emotional research in the field of entrepreneurship are Journal of Business Venturing (36), Frontiers in Psychology (24), Entrepreneurship Theory and Practice (23), International Entrepreneurship and Management Journal (15) and Journal of Small Business Management (15). In the top 10 journals, most of them are in the field of business and entrepreneurship, such as Journal of Business Venturing, Entrepreneurship Theory and Practice, and Journal of Business Research. As emotion in the area of entrepreneurship is an interdisciplinary issue, psychological journals such as Frontiers in Psychology also include a lot of related research.

**Table 1:** Top 10 journals in number of documents.

**Title**	**Documents**	**Rank**
Journal of Business Venturing	36	1
Frontiers in Psychology	24	2
Entrepreneurship Theory and Practice	23	3
International Entrepreneurship and Management Journal	15	4
Journal of Small Business Management	15	4
International Journal of Entrepreneurial Behavior Research	14	6
International Small Business Journal Researching Entrepreneurship	13	7
Sustainability	13	7
Journal of Business Research	12	9
Small Business Economics	10	10

### Country/Regional Distribution

According to the ranking of the number of posts and the number of citations of various countries (see Table 2), we find that the three countries with the largest number of articles are the United States (142), the United Kingdom (60), and China (56). The three countries with the most citations of published articles are the United States (10,978), the United Kingdom (2,431), and Germany (2,366). The number of emotional studies published in the United States in the field of entrepreneurship is far higher than other countries, with a total of more than 10,000 citations. American scholars attach great importance to the emotional research in the field of entrepreneurship and have published many influential achievements. In addition, Britain also has a high degree of attention and contribution to this field. It is worth noting that the vast majority of the countries on the list are developed countries, and China is the only developing country. It shows that compared with developing countries, developed countries have higher attention and more in-depth research in this field.

**Table 2:** Top 10 countries/regions in number of documents and citations.

**Country/region**	**Documents**	**Rank**	**Country/region**	**Citations**	**Rank**
USA	142	1	USA	10,978	1
Britain	60	2	Britain	2,431	2
China	56	3	Germany	2,366	3
Germany	41	4	Canada	1,430	4
Spain	39	5	Spain	1,367	5
Netherlands	33	6	Sweden	1,146	6
Australia	30	7	Netherlands	627	7
Canada	24	8	Finland	533	8
France	24	9	Australia	529	9
Sweden	23	10	China	397	10

### Co-authors Analysis

**Figure 2:**
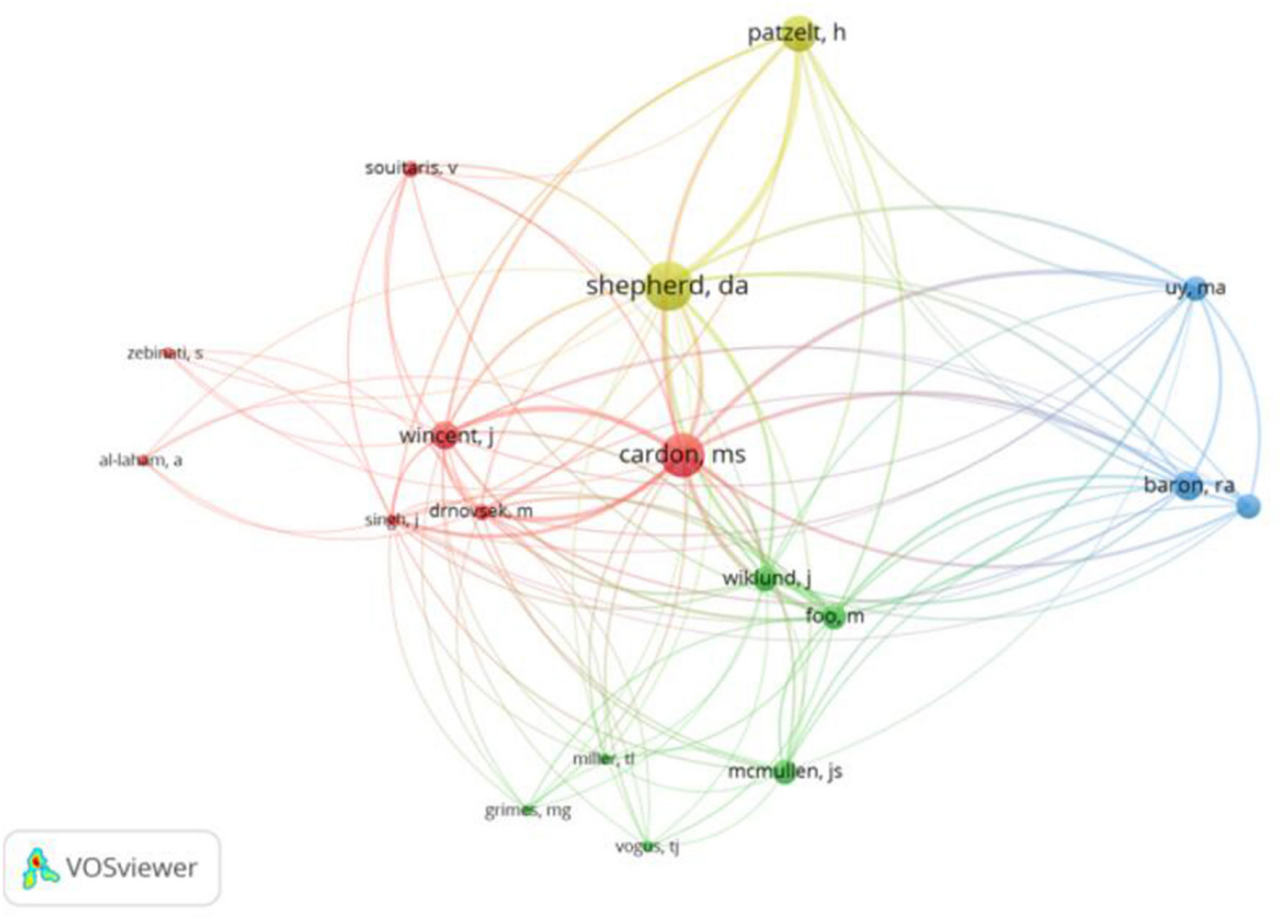
Authors cooperation network.

As shown in Table 3, we show the authors with the top 20 citations of published articles (including juxtaposition). The authors of the top three cited times are Cardon, MS (1,939), Shepherd, DA (1,926), Baron and RA (1,904). We use VOSviewer software to analyze the top 20 authors and get the author cooperation network graph (Figure 2). We find that there is a cooperative relationship between 18 of the 20 authors. It is worth noting that the authors Cardon and MS have worked with all 17 authors on the list, and many articles have a good influence. In addition, among the authors in the top 20 citations, Berrone, PJournal Cruz, C, and Gomez-mejia, LR co-published the most frequently cited article as co-authors, while Miller TL, Grimes MG and Vogus TJ also entered the list for co-authoring an article (see Table 4). According to the top ten most frequently cited articles shown in Table 4, we know that the topics of these influential articles are related to social emotional wealth, college students' entrepreneurial intention, entrepreneurial passion, entrepreneurial success or failure, and the impact of emotion on entrepreneurship and other topics.

**Table 3:** Top 20 cited authors (including juxtaposition).

**Author**	**Documents**	**Citations**	**Rank**
Cardon, MS	13	1,939	1
Shepherd, DA	16	1,926	2
Baron, RA	6	1,904	3
Berrone, P	1	976	4
Cruz, C	1	976	4
Gomez-mejia, LR	1	976	4
Souitaris, V	2	924	7
Al-laham, A	1	915	8
Zebinati, S	1	915	8
Foo, M	4	889	10
Patzelt, H	9	882	11
Hmieleski, KM	4	862	12
Wincent, J	5	790	13
Drnovsek, M	2	779	14
Singh, J	1	720	15
Mcmullen, JS	4	575	16
Uy, MA	4	433	17
Wiklund, J	4	432	18
Grimes, MG	1	431	19
Miller, TL	1	431	19
Vogus, TJ	1	431	19

**Table 4:** Top 10 articles cited.

**Author**	**Title**	**Journal**	**Citations**
Berrone, P Cruz, C Gomez-Mejia, LR	Socioemotional wealth in family firms: Theoretical dimensions, assessment approaches, and agenda for future research	Family Business Review	976
Souitaris, V Zerbinati, S Al-Laham, A	Do entrepreneurship programmes raise entrepreneurial intention of science and engineering students? The effect of learning, inspiration and resources	Journal of Business Venturing	915
Cardon, MS Wincent, J Singh, J Drnovsek, M	The nature and experience of entrepreneurial passion	Academy of Management Review	720
Baron, RA	Cognitive mechanisms in entrepreneurship: Why and when entrepreneurs think differently than other people	Journal of Business Venturing	664
Shepherd, DA	Learning from business failure: propositions of grief recovery for the self-employed	Academy of Management Review	485
Hmieleski,KM Baron, RA	Entrepreneurs' optimism and new venture performance: a social cognitive perspective	Academy of Management Review	451
Miller, TL Grimes, MG McMullen, JS Vogus, TJ	Venturing for others with heart and head: how compassion encourages social entrepreneurship	Academy of Management Review	431
Gatewood, EJ Shaver, KG Gartner, WB	A longitudinal-study of cognitive-factors influencing start-up behaviors and success at venture creation	Journal of Business Venturing	322
Foo, MD Uy, MA Baron, RA	How do feelings influence effort? An empirical study of entrepreneurs' affect and venture effort	Journal of Applied Psychology	310
Cardon, MS Zietsma, C Saparito, P Matherne, B P Davis, C	A tale of passion: new insights into entrepreneurship from a parenthood metaphor	Journal of Business Venturing	302

### Research Hotspot Analysis

**Figure 3:**
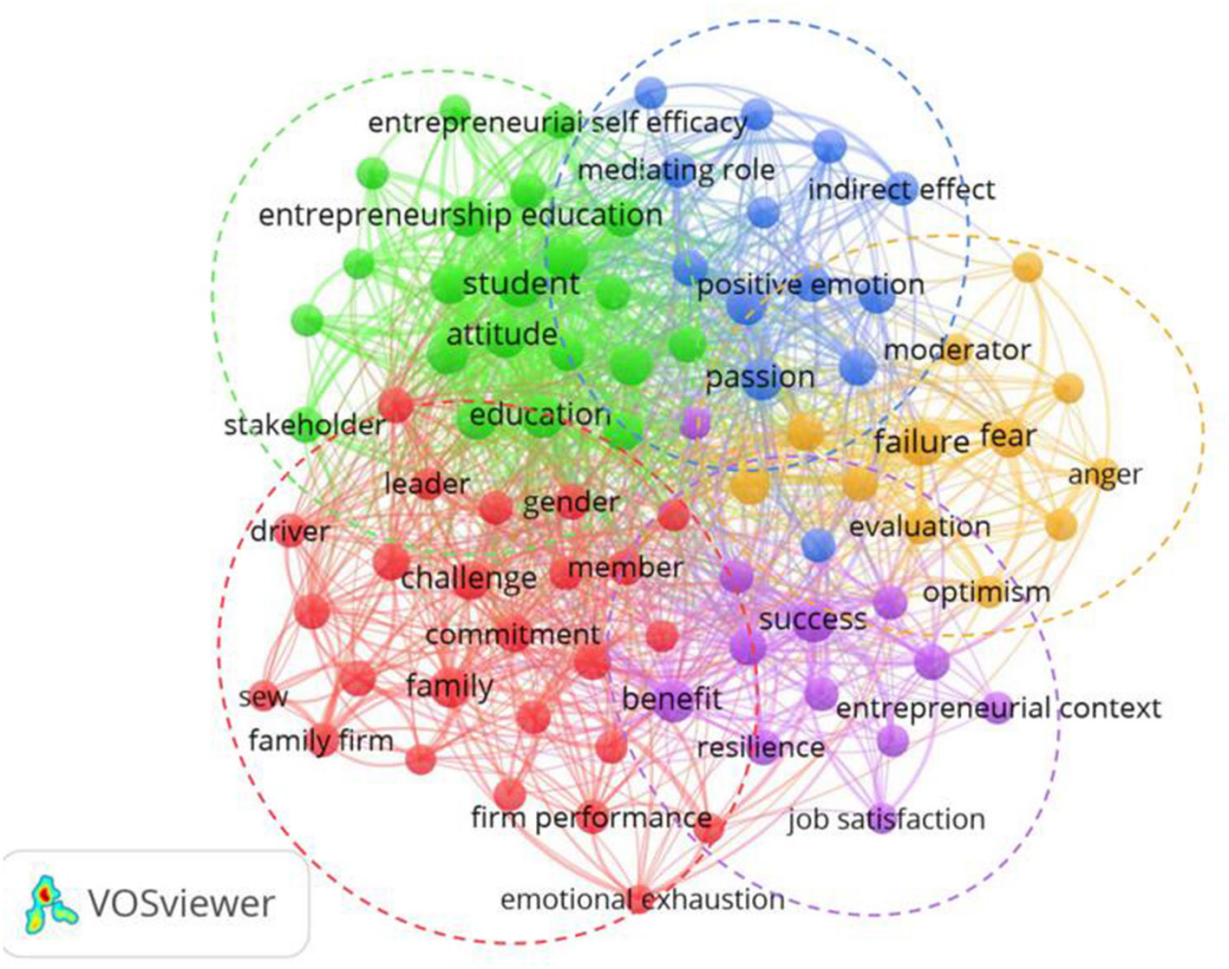
Research hotspot map.

We use VOSviewer software to analyze the abstracts of 479 articles and construct a term co-occurrence network diagram. In the abstracts of 479 articles, a total of 8,971 terms are extracted, and the terms appearing at least 8 times will appear in the term co-occurrence network diagram. Among these terms, we retained the top 60% of the most relevant terms and finally got 199 terms with the highest relevance. Through data cleaning, we removed meaningless terms and merged synonyms. In the term co-occurrence network diagram, a large node indicates that this term appears frequently, while a colored connection indicates that two terms appear simultaneously. At the same time, nodes with the same color indicate that the relevant terms may come from the same document. Therefore, we will read the documents related to the terms with the same color in full to understand what they focus on. Finally, according to the output of VOSviewer, the research on emotion in entrepreneurship focuses on five aspects (Figure 3).

## Cluster I: Emotion and College Students' Entrepreneurship

The terms in the green part of the research hotspot map shown in Figure 3 mainly include student, intention, entrepreneurship education, attitude, etc. Based on these terms, we generalized this group as emotion and college students' entrepreneurship. We found that the relevant research in this cluster focused on discussing the influence of emotion on college students' entrepreneurship and the relationship between entrepreneurship education and college students' entrepreneurial emotions.

### The Influence of Emotion on College Students' Entrepreneurship

Emotional competencies are the ability to feel, understand and effectively use the power of emotions (Goleman, 1998). Emotional intelligence and emotional competence are two related but different concepts. Emotional intelligence determines a person's potential to learn personal and social skills, while emotional competence indicates how much of this potential has been translated into application (Goleman and Cherniss, 2001). Many scholars have discussed the role of emotional competence of college students in entrepreneurship, and Fernandez-Perez et al. (2019) found that the development of emotional competence of college students facilitates entrepreneurship because emotional competence development directly affects the formation of entrepreneurial intentions. In addition to direct effects, there are also indirect effects of emotional competence on entrepreneurial intentions of college students. for example, self-management, social awareness and relationship management skills in emotional competence can have an impact on college students' entrepreneurial orientation and thus further influence entrepreneurial intentions (Padilla-Melendez et al., 2014).

Of course, emotional competence is not the only affective factor that influences entrepreneurship among college students. Studies have shown that mentorship (including emotional aspects) can help college students transition into entrepreneurship (Ahsan et al., 2018). In addition, emotional help from family also deserves attention, Edelman et al. (2016) surveyed college students from 19 countries and empirically showed that emotional support from family can promote college students' participation in entrepreneurial activities.

### Entrepreneurship Education and College Students' Entrepreneurial Emotions

Entrepreneurship education is an effective program to promote entrepreneurship among college students, and it can significantly increase the level of entrepreneurship (Jack and Anderson, 1998). Studies have shown that entrepreneurship education can influence students' mindset by affecting their emotions, thus motivating them to develop a more entrepreneurial mindset (Cui et al., 2021). In addition, entrepreneurship education can also enhance entrepreneurial intentions by regulating students' emotions such as passion and optimism (Haddoud et al., 2022). The importance of emotional competence is well understood, and in fact, students with higher emotional competence have more positive attitudes toward entrepreneurship and consider themselves more capable of becoming entrepreneurs after receiving entrepreneurship education.

As part of entrepreneurship education, educators also have an important role. Studies have found that when students are emotionally positive, guidance from teachers leads them to successfully launch their own businesses (Ahsan et al., 2018). In addition, educators also have a very important role to play for students emotionally depressed. Negative emotions have a significant impact on college students' entrepreneurship, but educators can combine some negative emotions such as students' dissatisfaction with their current employment situation with motivating students' entrepreneurial intentions (Wang et al., 2016). Teachers can also give guidance to help students successfully redirect their entrepreneurship when their emotions are negative (Ahsan et al., 2018).

However, in addition to entrepreneurship education, universities should provide emotional education to potential entrepreneurs to equip them with emotional adaptation skills to overcome the challenges of entrepreneurship (Aly et al., 2021). In addition, experiential learning practices (e.g., outdoor training) can help improve the emotional competence of college students (Hamilton and Cooper, 2001), and colleges can implement experiential learning programs such as outdoor training to equip students with the ability to cope with emotional challenges while developing their entrepreneurial intentions by improving their emotional competence (Padilla-Melendez et al., 2014). In addition, blended entrepreneurial program is an effective educational strategy. Unlike traditional entrepreneurship education, it is a program that combines an entrepreneurship curriculum with a technical degree (Kuratko, 2005). Research has shown that combining blended entrepreneurial programs with students' entrepreneurial enthusiasm can effectively increase the likelihood that students develop entrepreneurial intentions (Turner and Gianiodis, 2018).

## Cluster II: Family Emotions and Entrepreneurship

The terms in the red part of the research hotspot map include family, family firm, sew, emotional attachment, emotional support, etc. This cluster can be summarized as family emotions and entrepreneurship. Combining these terms, after extensive reading of relevant literature, we think that the existing studies on family emotion and entrepreneurship are mainly discussed from the following two aspects: the role of emotions on entrepreneurship in family firms, and the influence of family emotional support on entrepreneurial activity.

### The Role of Emotions on Entrepreneurship in Family Firms

The boundary between family and business is very blurred in family firms, so emotions can permeate family firms and influence decision making (Baron, 2008; Berrone et al., 2010, 2012), that's why there is a clear difference between family and non-family firms.

Socioemotional wealth (SEW) can help explain the difference between family and non-family firms (Gomez-Mejia et al., 2007), representing a set of non-economic goals that satisfy the emotional needs of family members (Gjergji et al., 2022), such as family legitimacy, family image, family harmony or employment of family members (Gomez-Mejia et al., 2007; Howorth et al., 2010; Berrone et al., 2012; Debicki et al., 2016; Munoz-Bullon et al., 2020). Studies have shown that family firms are less entrepreneurially oriented than non-family firms (Garces-Galdeano et al., 2016), as the desire to protect family wealth can lead family firm managers to be too conservative in the face of entrepreneurial risk (Zahra, 2005; Naldi et al., 2007). However, some scholars believe that it is inappropriate to study the impact of SEW on entrepreneurship only as a whole. SEW has five dimensions (FIBER) (Berrone et al., 2012), and some scholars believe that different dimensions of SEW have different effects on family business entrepreneurship, some dimensions may have positive effects on entrepreneurship, while others may have negative effects or Some dimensions may have a positive impact on entrepreneurship, while others may have a negative impact or no significant impact (Filser et al., 2018).

Emotional attachment, as one of the five dimensions of SEW reflecting emotional aspects, deals with the role of emotions in family business (Berrone et al., 2012). Emotional attachment may have an impact on family members' decision making and thus on family business entrepreneurship (Gjergji et al., 2022). On the one hand, emotional attachment may promote creativity development and opportunity recognition and thus entrepreneurship; on the other hand, emotional attachment makes family business managers tend to protect the welfare of family members and stick to the enterprise's initial strategy, thus preventing family members from pursuing risky opportunities and stopping the family business (Aldrich and Cliff, 2003; Kellermanns et al., 2012; de Massis et al., 2015). Therefore, managers of family businesses need to improve their professional competencies by improving their education to cope with the range of challenges that emotions pose to the business (Pellegrini and Lazzarotti, 2019).

### The Influence of Family Emotional Support on Entrepreneurial Activity

Entrepreneurial activity in family firms is not the only one that is influenced by family, but general entrepreneurship is also influenced by family. Studies have shown that a supportive and encouraging family environment is more conducive to entrepreneurial activity than a distant and unwelcoming family environment (Baughn et al., 2006; Essers and Benschop, 2009; Basco, 2015). Emotional support has a crucial role in the early stages of entrepreneurship (Bruderl and Preisendorfer, 1998; Klyver et al., 2018), emotional support includes listening, encouraging, understanding and caring (Powell and Eddleston, 2017; Klyver et al., 2018; Cogan et al., 2022). Family members help entrepreneurs to start and grow their businesses by providing emotional support and effectively cope with the various negative emotions (e.g., self-doubt, stress, and anxiety) that entrepreneurs face during the entrepreneurial process so that they have more confidence, motivation, and comfort (Cogan et al., 2022). In addition, for young entrepreneurs, emotional support from family can significantly contribute to the occurrence of entrepreneurial activities (Edelman et al., 2016).

## Cluster III: The Role of Emotions on Successful Entrepreneurship

The terms involved in the purple part of the research hotspot map include success, benefit, job satisfaction, resilience, etc. This part focuses on the role of emotions in successful entrepreneurship. We analyze the role of emotional intelligence and two emotions on successful entrepreneurship respectively.

### The Role of Emotional Intelligence

Research has shown that founders of successful startups exhibit higher levels of emotional intelligence (Cross and Travaglione, 2003). Emotional intelligence is the ability to identify, facilitate, and understand one's own emotions and their emotions (Mayer and Salovey, 1997), and it is important for managing stress and emotional breakdown (Slaski and Cartwright, 2002; Tsaousis and Nikolaou, 2005). Compared to mature firms, entrepreneurship research on emotional intelligence is less frequent (Andreea et al., 2014), and in fact emotional intelligence can have an impact on entrepreneurial intentions, entrepreneurial attitudes, etc. (Tiwari et al., 2017), and in addition it has profound implications for entrepreneurial success.

Emotional intelligence consists of two different structures, intrapersonal emotional intelligence and interpersonal emotional intelligence (Cherniss, 2010). Ingram et al. (2019) demonstrated that both types of entrepreneurial emotional intelligence are beneficial for entrepreneurial performance and success. They found that interpersonal emotional intelligence of entrepreneurs can make individuals and organizations function better and thus improve performance; while personal emotional intelligence indirectly helps firms achieve higher performance. Then Allen et al. (2021) used meta-analysis to investigate the effect of general mental ability and Emotional intelligence on entrepreneurial success and showed that emotional intelligence had a more significant positive effect on entrepreneurial success than general mental ability.

### The Role of Positive and Negative Emotions

In addition to emotional intelligence, both positive and negative emotions can affect entrepreneurial success. Many scholars believe that positive emotions positively affect entrepreneurial success (e.g., Baron and Tang, 2011; Dijkhuizen et al., 2018), while negative emotions are detrimental to entrepreneurial success (e.g., Diener et al., 2003; Bernoster et al., 2020). For example, Dijkhuizen et al. (2018) explored the relationship between positive emotions and performance and found that achieving increased entrepreneurial wellbeing was beneficial to entrepreneurial success, while Bernoster et al. (2020) investigated sole proprietors in the Netherlands and France and found a negative relationship between negative emotions and entrepreneurial success. However, it has also been argued that positive emotions as a signal that the business is going well can cause founders to become complacent and thus negatively affect the performance of the new venture (e.g., Hmieleski and Baron, 2009); whereas negative emotions contribute to vigilance and thus entrepreneurial success (e.g., Foo et al., 2009). On the other hand, Fodor and Pintea (2017) argued that negative emotions as states are negatively associated with entrepreneurial success, while negative emotions as traits are not associated with entrepreneurial success. In addition, high emotional stability plays an important role in entrepreneurial success (Hachana et al., 2018).

## Cluster IV: A Study of Emotional Problems Under the Influence of Entrepreneurial Failure

The orange part of the research hotspot map includes terms such as failure, fear, negative emotion, moderator, etc. We summarize this part of the content as the study of emotional problems under the influence of entrepreneurial failure. At present, there are few emotional studies related to entrepreneurial failure, but we review the relevant literature and think that such studies mainly discuss the emotional consequences, emotional resilience and emotional recovery related to entrepreneurial failure.

### Emotional Consequences of Entrepreneurial Failure

Some studies suggest that the consequences of entrepreneurial failure may be negative or positive. Ucbasaran et al. (2009) found that entrepreneurs are motivated to increase their efforts when the percentage of failure is small, but when the percentage of failure exceeds a certain point, it reduces the motivation of entrepreneurs to identify future opportunities. Fisch and Block (2021) argue that on the negative side, entrepreneurs become fearful of the unknown future after failure; on the positive side, entrepreneurs become more reflective after failure.

However, more scholars have studied the significant negative consequences of entrepreneurial failure- negative emotions. Studies have found psychological costs associated with entrepreneurial failure (Ucbasaran et al., 2013). For example, many negative emotions (self-blame, shame, anger, fear, etc.) are associated with entrepreneurial failure (Singh et al., 2007; Cope, 2011). In fact, negative emotions associated with entrepreneurial failure do not necessarily have negative effects; these negative emotions often have a dual function (Shepherd, 2009), while they impede learning behavior by competing for cognitive resources, at the same time these negative emotions can motivate learning behavior by reminding of mistakes. He et al. (2018), on the other hand, found through an empirical study that the relationship between failure rate and learning behavior is inverted U-shaped, i.e., learning behavior increases with failure rate until an inflection point and then decreases with increasing failure rate. However, they found that for entrepreneurs with high levels of emotional regulation, learning behavior still increased when the failure rate rose beyond the inflection point.

### Entrepreneurial Emotional Resilience

Emotional resilience is crucial to entrepreneurship, and resilient firms are more likely to succeed (Chadwick and Raver, 2020). In fact, entrepreneurial resilience also plays an extremely prominent role after entrepreneurial failure. Lafuente et al. (2019) found that resilient entrepreneurs are able to overcome negative emotions and start up again.

In addition, scholars have found that entrepreneurial failure helps to develop entrepreneurial resilience. Corner et al. (2017) explored the emotional and psychological functioning of entrepreneurs after entrepreneurial failure, and they found that most of the study participants demonstrated entrepreneurial resilience (stability in emotional and psychological functioning), while prior failures may build resilience for subsequent ventures. Lafuente et al. (2019), on the other hand, found that that these resilient serial entrepreneurs created firms with a better international orientation compared to nascent entrepreneurs. At the same time, it has been shown that self-compassion resulting from loving-kindness meditation can also help entrepreneurs develop resilience and thus cope with their fear of failure (Engel et al., 2021). In addition, it has been found that high levels of self-confidence also contribute to entrepreneurial resilience, as more confident entrepreneurs experience greater emotional resilience from failed ventures (Hayward et al., 2010).

### Emotional Recovery From Entrepreneurial Failure

Shepherd (2009) suggests that entrepreneurs with better emotional intelligence are able to understand failure more effectively and recover quickly from it. In addition to the entrepreneurial resilience and emotional intelligence of founders that contribute to recovery from entrepreneurial failure, the level of self-confidence also plays an important role, as self-confidence reduces the emotional cost of failure and allows entrepreneurs to experience fewer negative emotions (Hayward et al., 2010). In addition, Jenkins et al. (2014) found that entrepreneurial failure can cause entrepreneurs to feel a loss of self-esteem, so rebuilding self-esteem can help entrepreneurs alleviate the emotional toll of entrepreneurial failure; also, entrepreneurial failure and unemployment have similar strong emotions, so providing support to failed businesses analogous to unemployment benefits can also alleviate the negative emotions of entrepreneurs to some extent. Patzelt et al. (2021), on the other hand, realized that in addition to entrepreneurs, employees' negative emotions after entrepreneurial failure should also be taken into account, and they argued that in addition to fostering an organizational climate that accepts failure, managers should support employees who have negative emotions; furthermore, they found that supportive leaders can help employees alleviate negative emotions after entrepreneurial failure shortly after failure and thus improve employees' job satisfaction and performance.

## Cluster V: Entrepreneurial Passion

The terms in the blue part of the research hotspot map mainly include passion, positive emotion, positive effect, indirect effect, etc. Therefore, this cluster focuses on the research on entrepreneurial passion. These studies on entrepreneurial passion find that entrepreneurial passion has effects on entrepreneurs, employees and investors.

Entrepreneurial passion refers to the conscious, strong positive emotions experienced when engaging in entrepreneurial activities, and it can have an impact on entrepreneurs (Cardon et al., 2009).

Some scholars have studied the impact of entrepreneurial passion on entrepreneurs, for example, Ruskin et al. (2016) explored social entrepreneurial motivation, and they found that entrepreneurial passion leads to self-directed social entrepreneurial motivation. Feng and Chen (2020), on the other hand, constructed a model of the relationship between entrepreneurial passion and entrepreneurial psychology and behavior based on self-efficacy theory, and found that entrepreneurial passion can increase entrepreneurial persistence and enhance firm performance by stimulating positive emotions in entrepreneurs. Of course, different types of entrepreneurial passion may have different effects on entrepreneurs, with some studies suggesting that entrepreneurs passionate about products prioritize their decisions based on the effects principle, while entrepreneurs passionate about growth rely mainly on causal logic (Cannatelli et al., 2019).

Cardon (2008) showed that passionate entrepreneurs are more likely to engage in transformational leadership with their employees, and that transformational leadership enhances an organization's perception of its employees by strengthening the social comparison process, the alignment of employees with organizational goals, and the employees' sense of team transformational leadership enhances the meaning of the organization to the employees by strengthening the social comparison process, the alignment of employees with organizational goals, and the employees' sense of identity with the team, thereby transferring the entrepreneurial passion of the entrepreneur to the employees. Later scholars have also confirmed that entrepreneurial passion is contagious (e.g., Mitteness et al., 2012; Davis et al., 2017). Hubner et al. (2020) developed a mediation model and found through empirical studies that entrepreneurs can use their entrepreneurial passion to motivate employees' passion response, commitment, and performance: entrepreneurs can make employees passionate about entrepreneurial activities by communicating their passion to them, and their passion has a particularly strong effect on employees those are not passionate about entrepreneurial activities, so their passion can compensate for their lack of passion. Cardon et al. (2009) concluded that there were three types of entrepreneurial passion, namely, passion for invention, entrepreneurship, and development. In contrast, Breugst et al. (2012) found that entrepreneurial passion for invention, entrepreneurship and development have different effects on employee commitment. Entrepreneurs' enthusiasm for invention and development increases commitment, but enthusiasm for entrepreneurship weakens commitment.

In addition, entrepreneurial passion has a significant role in obtaining investment in entrepreneurial firms. Using a combination of qualitative and quantitative research methods, Murnieks et al. (2016) found that angel investors value entrepreneurial passion in addition to entrepreneurial resilience when evaluating investments. And Davis et al. (2017) confirmed through empirical tests that financing propositions made by entrepreneurs perceived as highly passionate do have greater impact.

## Future Research

**Figure 4:**
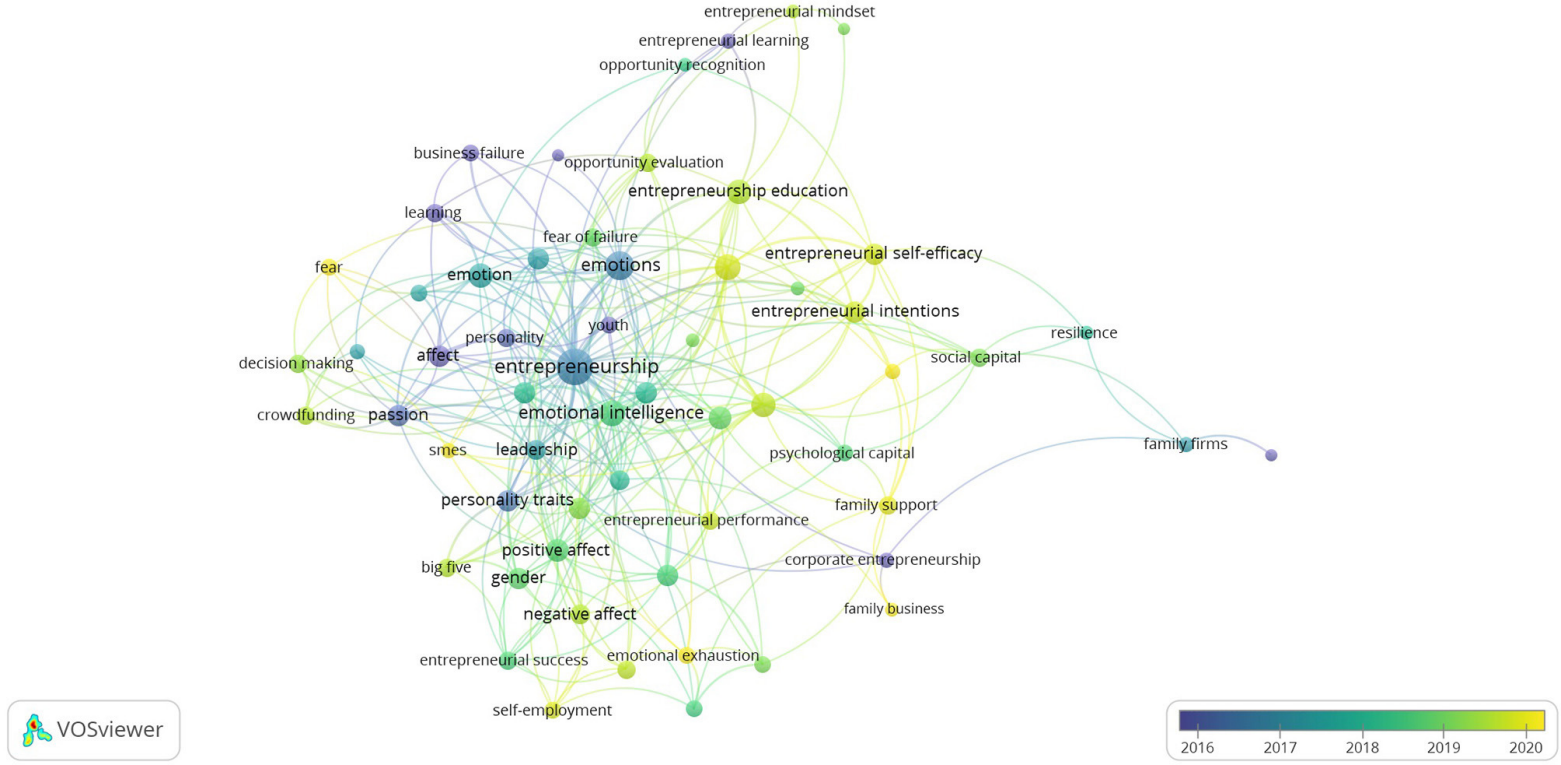
Future research.

In this study, VOSviewer software is used to show the time series of the author keywords in emotional research in the area of entrepreneurship. By observing the time series, we can clearly and intuitively understand the research trends in this field. In Figure 4, the color of the keyword node represents the year when these keywords first appear. The keyword with blue node color appears in the earliest year, while the keyword with yellow node color appears in the latest year, and the corresponding research content is also more cutting-edge.

Through the analysis of emerging keywords and corresponding research, we found that the existing research focuses on the relationship between emotion (including positive or negative emotion, family emotion, emotional intelligence or competency, etc.) and entrepreneurship (including entrepreneurial attitude or intention, entrepreneurial opportunity identification, college students' entrepreneurship, entrepreneurial success or failure, family business entrepreneurship, etc.). These studies have important reference value and enlightening significance for the follow-up research, but there are still some deficiencies or omissions in these studies. Therefore, we propose the following future research directions for reference, and scholars can further improve the research in these directions.

### Research on the Impact of Specific Emotion on Entrepreneurship

By combing the emotional studies in the area of entrepreneurship, we found that there are a large number of studies focusing on the impact of emotion on entrepreneurship, but these studies mainly discuss the role of positive emotion or negative emotion. In fact, two specific emotions, both positive (or negative), may have different effects on entrepreneurship. For example, entrepreneurial passion and confidence are both positive emotions. However, entrepreneurial passion has a positive impact on entrepreneurship (Feng and Chen, 2020), while self-confidence is sometimes detrimental to entrepreneurship (Hayward et al., 2010). Obviously, it is not reasonable to classify emotion into positive emotion or negative emotion to study its impact on entrepreneurship. Although some scholars have noticed the impact of specific emotions (such as passion, fear, satisfaction, etc.) on entrepreneurship, these studies are still inadequate. Future research should focus more on the impact of specific emotions such as fear of failure, emotional exhaustion and desire for success on entrepreneurship.

### Research on the Effects of Employee Emotion on Entrepreneurship

At present, the emotion research in the area of entrepreneurship mostly focuses on the emotion of entrepreneurs, and there are few studies on the emotion of employees. In fact, although employees do not start companies, they are important participants in entrepreneurial activity, so enhancing research on the emotions of start-up employees is crucial. Some scholars have noticed the emotion of employees in starting a business. For example, some scholars have found that entrepreneurs can transfer their passion to employees (e.g., Breugst et al., 2012), and employees' passion can further affect starting a business (e.g., Hubner et al., 2020). However, research in this area is obviously not enough. Understanding the relationship between employee emotion and entrepreneurship can help entrepreneurs make better use of employees' potential. Therefore, more attention can be paid to the connection between employees' emotions and entrepreneurship in the future.

### Research on Emotion in Various Entrepreneurial Behaviors

At present, some studies focus on the emotional problems in specific situations such as female entrepreneurship, family business entrepreneurship and college students' entrepreneurship (e.g., Berrone et al., 2012; Edelman et al., 2016; Welsh et al., 2021). These emotional studies on specific types of entrepreneurship can help scholars understand the impact and function of emotion in different situations. With the continuous enrichment of emotional research in the area of entrepreneurship, future research has a more solid theoretical basis and empirical basis. Therefore, we suggest that future scholars supplement relevant research, such as in-depth research on the emotional content of underdog entrepreneurship, sustainable entrepreneurship, digital entrepreneurship and other types of entrepreneurship.

### Research on the Role of Emotional Support in Entrepreneurship

Entrepreneurship is an activity rooted in society (Downing, 2005), and social support even plays a decisive role in the critical period of entrepreneurship (Klyver et al., 2018). As a part of social support, emotional support is of great significance to entrepreneurship. Some scholars have noticed the significance of emotional support as an emotional factor in entrepreneurship. For example, some scholars have confirmed the role of family emotional support in promoting entrepreneurship (e.g., Edelman et al., 2016; Cogan et al., 2022). Entrepreneurial activities of entrepreneurs are often accompanied by financial risks and psychological pressure, and emotional support from family or society can help entrepreneurs to better make decisions or learn under such circumstances. At present, more and more scholars are paying attention to the topic of emotional support. We suggest that future research deeply analyze the role of emotional support in each stage of entrepreneurship and discuss the role of emotional support for different entrepreneurs. At present, more and more scholars are paying attention to the topic of emotional support. We suggest that future research deeply analyze the role of emotional support in each stage of entrepreneurship and discuss the role of emotional support for different entrepreneurs.

## Conclusion

In this study, we systematically reviewed the emotional research in the area of entrepreneurship. We collected 479 articles from the Web of Science and analyzed them using VOSviewer software.

First of all, we found that the number of emotional research in the area of entrepreneurship has increased year by year. At the same time, the journals that publish research on emotions in entrepreneurship are mainly management journals, and some psychology journals also publish such research. At present, most of the countries that pay attention to this field are developed countries, and the research in these countries has more influence than that in developing countries. It is worth noting that there is a close cooperative relationship among the influential authors in the emotional research in the area of entrepreneurship, and they jointly published insightful research.

Secondly, we use VOSviewer to conduct term co-occurrence analysis. We analyzed the existing research on emotions in entrepreneurship and found that the current research hotspots can be divided into five categories: emotions and entrepreneurship among college students, family emotions and entrepreneurship, the role of emotions in successful entrepreneurship, emotions in the context of entrepreneurial failure, and entrepreneurial passion.

Thirdly, we analyze the time series of keywords with the help of VOSviewer, and we propose several future research directions for reference. We believe that future research can deepen these directions: research on the impact of specific emotion on entrepreneurship, research on employee emotion and entrepreneurship, research on emotion in various entrepreneurial types, research on the role of emotional support in entrepreneurship.

This research scientifically and comprehensively analyzes the past, present and future of emotional research in the area of entrepreneurship. This study provides support for subsequent researchers and inspiration for universities and entrepreneurs.

## Data Availability Statement

The original contributions presented in the study are included in the article/supplementary material, further inquiries can be directed to the corresponding author/s.

## Author Contributions

All authors listed have made a substantial, direct, and intellectual contribution to the work and approved it for publication.

## Funding

This research was partly supported by the National Natural Science Foundation of China [Nos. 72002080 and 72172052] and Key Topics of Heilongjiang Education Science Planning [Nos. GBB1317093 and GJB1421031].

## Conflict of Interest

The authors declare that the research was conducted in the absence of any commercial or financial relationships that could be construed as a potential conflict of interest.

## Publisher's Note

All claims expressed in this article are solely those of the authors and do not necessarily represent those of their affiliated organizations, or those of the publisher, the editors and the reviewers. Any product that may be evaluated in this article, or claim that may be made by its manufacturer, is not guaranteed or endorsed by the publisher.
